# Supplementary material for: Augmentation of vaccine-induced humoral and cellular immunity by a physical radiofrequency adjuvant
Source: Nat Commun. 2018 Sep 12;9:3695. doi: 10.1038/s41467-018-06151-y (PMC6135850; doi:10.1038/s41467-018-06151-y)
Supplement: Supplementary file 1 — Supplementary Information [file 41467_2018_6151_MOESM1_ESM.pdf]

Augmentation of vaccine-induced humoral and cellular immunity by a physical radiofrequency adjuvant

Cao et al.

Supplementary Information

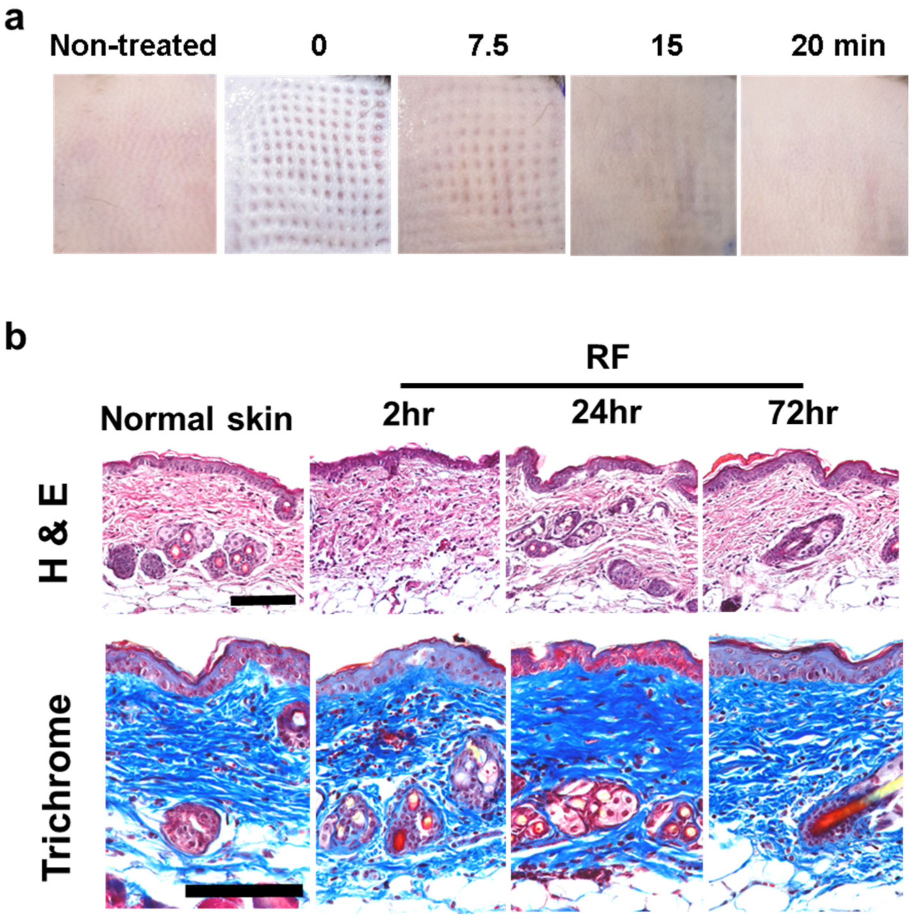

Supplementary Figure 1. RF treatment (1.5 minutes) induces minimal local reactions. (**a**) RF tips were firmly pressed on lateral back skin of C57BL/6 mice and held still for 1.5 minutes (min) for RF treatment. Representative skin pictures taken before or at different time points after RF treatment. (**b**) Lateral back skin of C57BL/6 mice was exposed to 1.5 min of RF treatment. RF-treated skin was dissected at different time points and subjected to paraffin sectioning and H & E staining to evaluate microscopic skin damages (upper panels) or trichrome staining to evaluate dermal collagen levels (lower panels). Representative H & E and trichrome-stained pictures were shown. Scale: 100μm (both). Representative of three independent experiments.

a

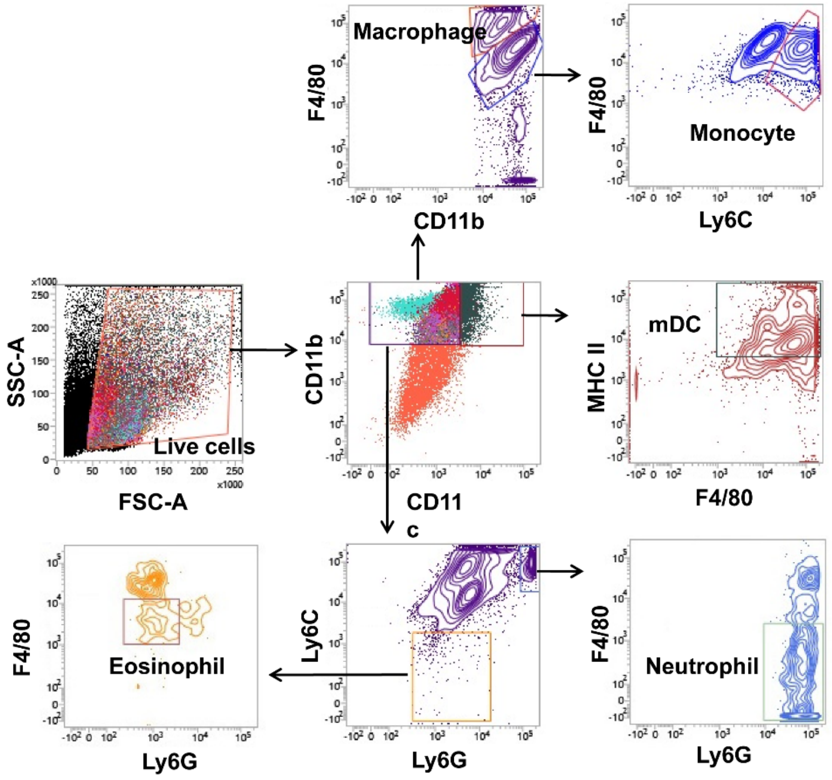

b

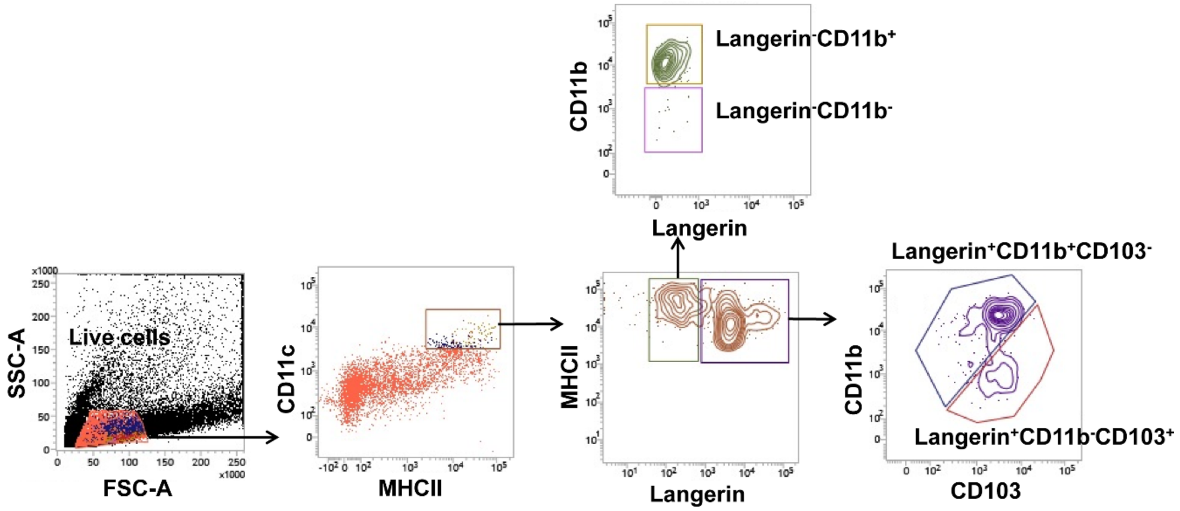

(Continued on the next page)

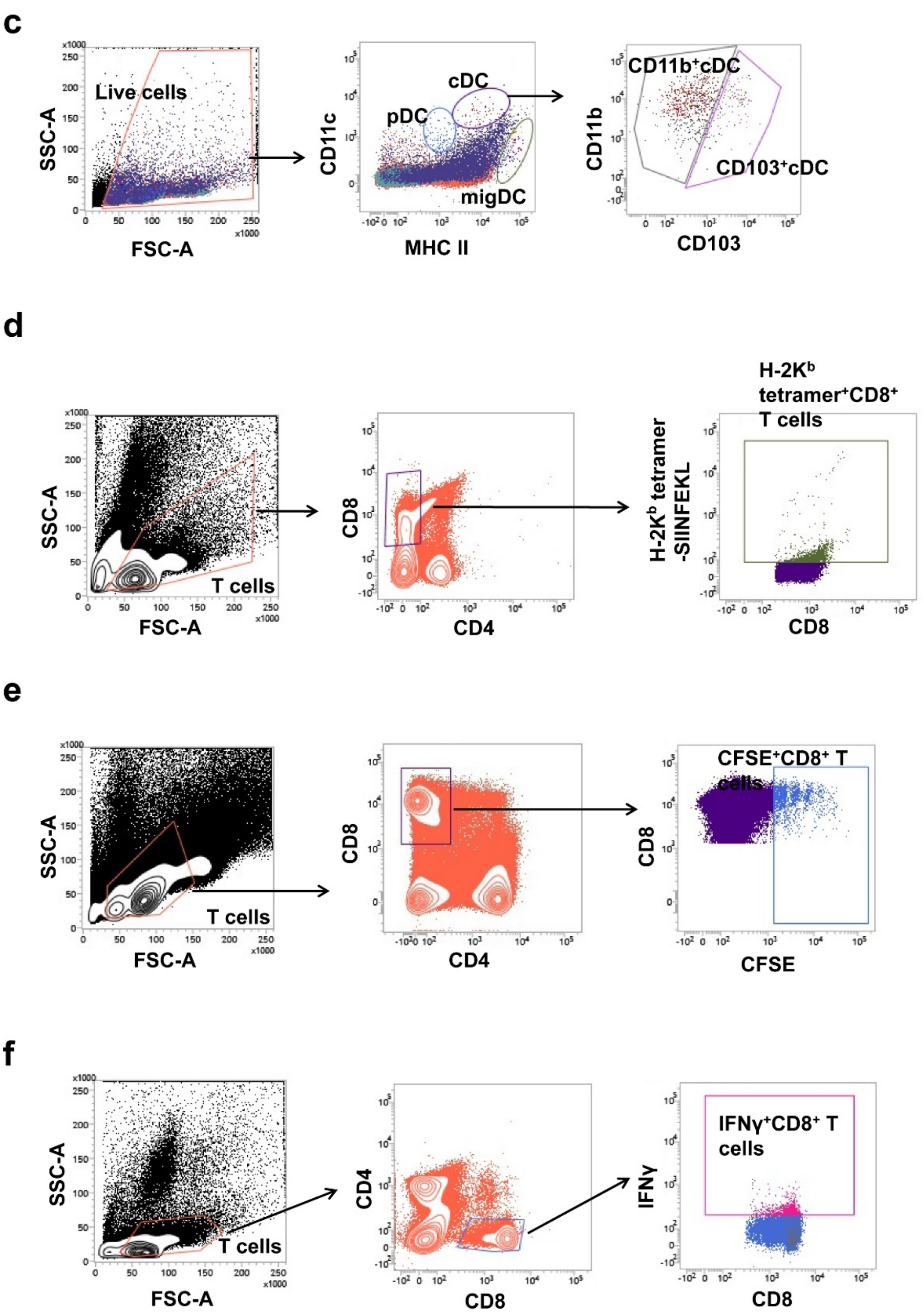

Supplementary Figure 2. Gating strategies used in cell analysis. (**a**) Gating strategy to identify different innate immune cells in Fig. 1c-g. (**b**) Gating strategy to identify DC subsets in skin (Fig. 2). (**c**) Gating strategy to identify DC subsets in dLNs (Fig. 3). (**d**) Gating strategy to evaluate tetramer$^+$ CD8$^+$ T cells in PBMCs in Fig. 4b, c. (**e**) Gating strategy to analyze proliferation of CFSE$^+$ OT-I cells in dLNs in Fig. 6. (**f**) Gating strategy to analyze IFNγ-secreting CD8+ T cells in PBMCs in Fig. 7d, e. The same strategy was also used to analyze IFNγ- and IL4-secreting CD8+ T cells in splenocytes in Fig. 5d, e.

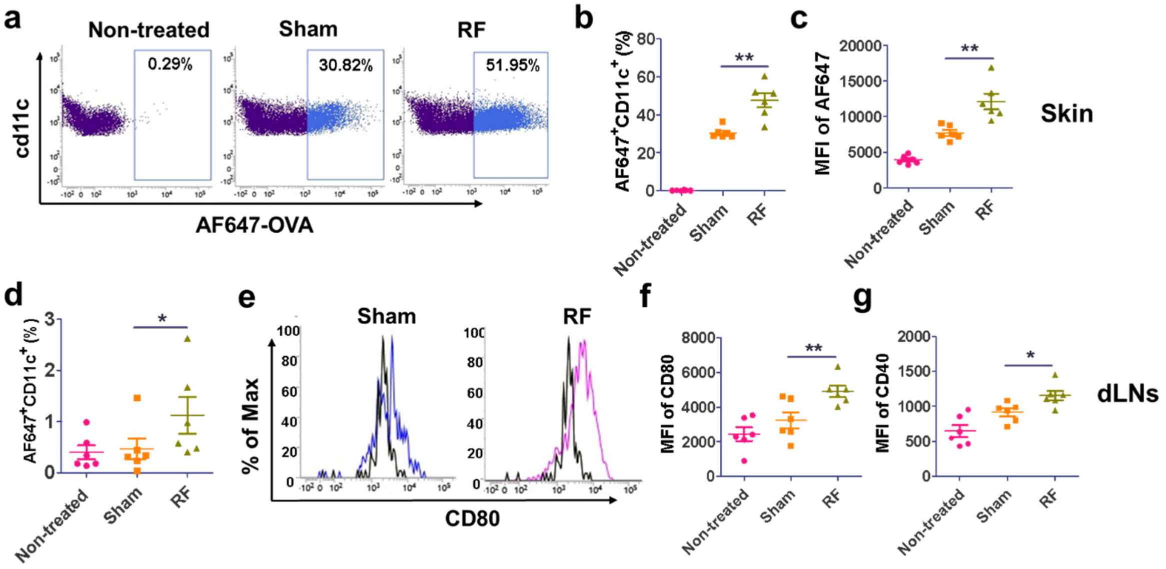

Supplementary Figure 3. RF increases antigen uptake and maturation of DCs. Lateral back skin of C57BL/6 mice was exposed to RF or sham treatment followed by ID injection of 2μg AF647-OVA into RF or sham-treated skin. (**a-c**) Skin was dissected 18 hours later and digested with collagenase D and dispase to prepare single-cell suspensions. Cells were then stained with fluorescence-conjugated antibodies against CD11c followed by flow cytometry analysis of percentage of AF647$^+$CD11c$^+$ cells. Cells were first gated based on FSC and CD11c and CD11c$^+$ cells were then analyzed based on AF647 levels. (**a**) Representative dot plots showing the percentage of AF647$^+$CD11c$^+$ cells. (**b**) Percentage of AF647$^+$CD11c$^+$ cells of different groups. (**c**) MFI of AF647 in AF647$^+$CD11c$^+$ cells. (**d-g**) dLNs were collected 18 hours after injection and passed through cell strainers to prepare single-cell suspensions. Cells were than stained with fluorescence-conjugated antibodies against CD11c, CD40, CD80, and CD86 followed by flow cytometry analysis of percentage of AF647$^+$CD11c$^+$ cells and MFI of CD40, CD80, and CD86. (**d**) Percentage of AF647$^+$CD11c$^+$ cells of different groups. (**e**) Representative histograms showing MFI of CD80 in AF647$^+$CD11c$^+$ cells. Black line: non-treated; blue line: sham; purple line: RF. (**f**) MFI of CD80 in AF647$^+$CD11c$^+$ cells. (**g**) MFI of CD40 in AF647$^+$CD11c$^+$ cells. n=6. Student's t-test was used to compare differences between groups. *, $p<0.05$; **, $p<0.01$. Representative of two independent experiments.

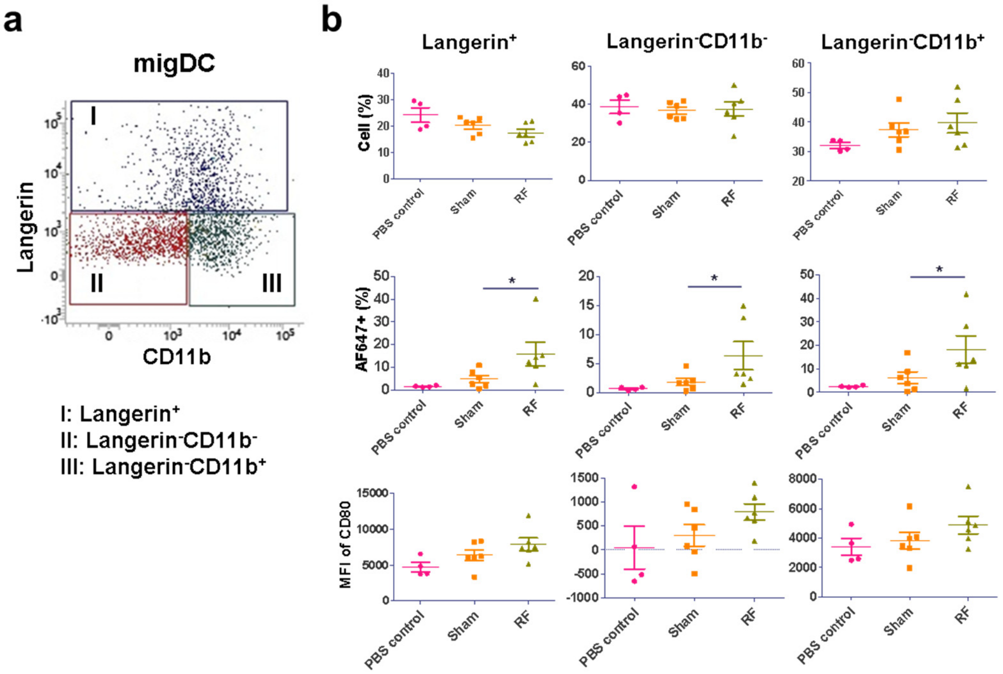

Supplementary Figure 4. RF increases antigen uptake of all migDC subsets in dLNs. migDC in Fig. 3 was further gated into 3 subsets based on expression of Langerin and CD11b (**a**). Percentage of each migDC subset (upper panels), percentage of AF647$^+$ cells (middle panels), and MFI of CD80 (lower panels) were then analyzed (**b**). n=4 for PBS control and 6 for Sham and RF groups. Student's t-test was used to compare differences between Sham and RF groups in B. *: $p<0.05$. Representative of two independent experiments.

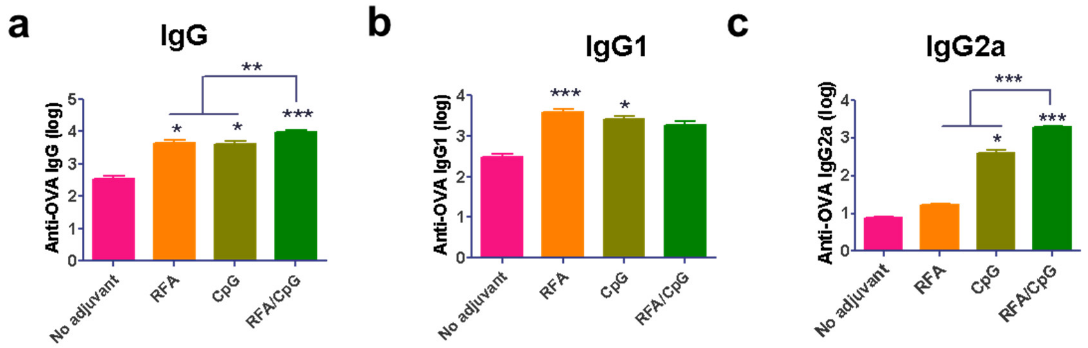

Supplementary Figure 5. RF induces Th2-biased immune responses in BALB/c mice. Lateral back skin of BALB/c mice was exposed to RF or sham treatment followed by ID injection of 10µg OVA into RF or sham-treated skin in the presence or absence of 30µg CpG. Serum anti-OVA IgG (**a**) and subtype IgG1 (**b**) and IgG2a antibody titer (**c**) was measured 2 weeks after immunization. n=4-6. One-way ANOVA with Tukey's multiple comparison test was used to compare differences between groups. *, $p<0.05$; **, $p<0.01$; ***, $p<0.001$.

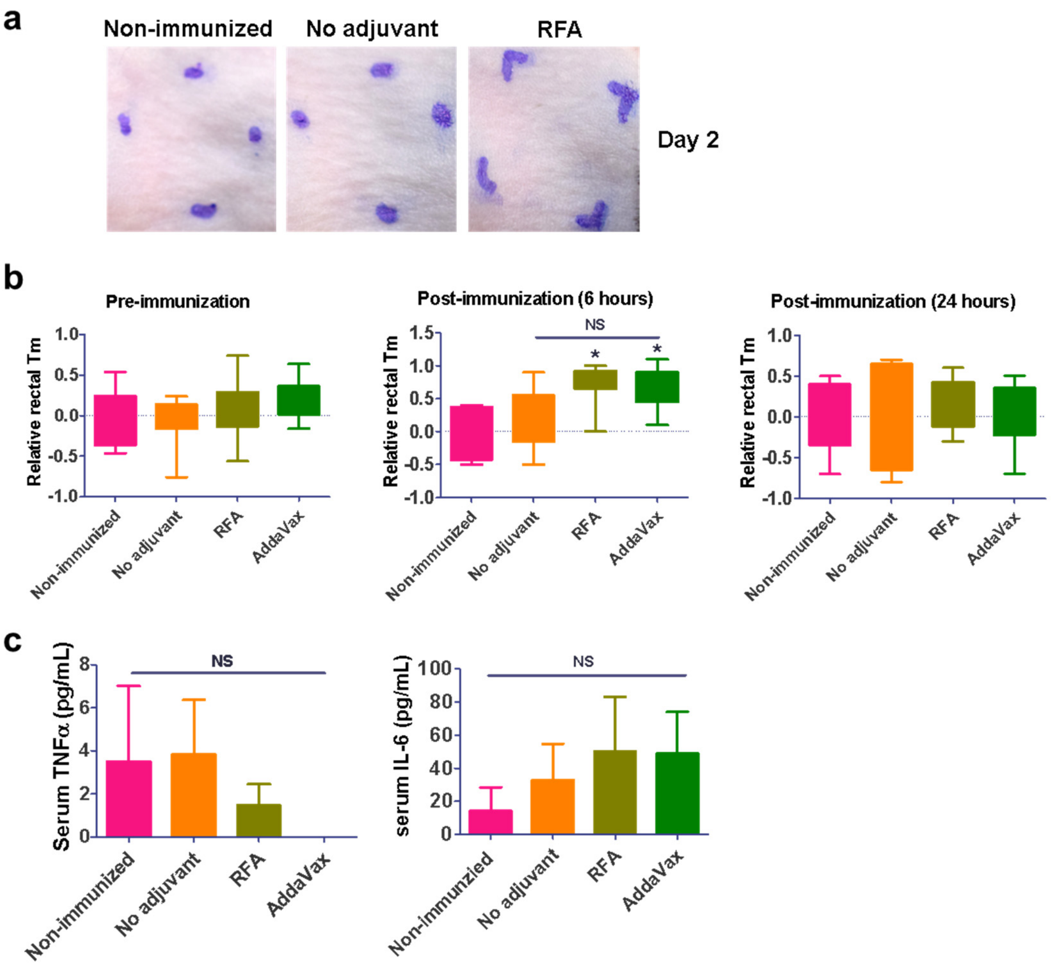

Supplementary Figure 6. Pdm09 vaccination in the presence of RFA induces minimal local and systemic reactions. C57BL/6 mice were intradermally immunized with 0.3μg pdm09 vaccine alone (no adjuvant) or in the presence of RFA (RFA), or intramuscularly immunized with the same vaccine dose in the presence of AddaVax (AddaVax), or intradermally injected with the same volume of PBS (non-immunized). (**a**) Skin pictures were taken 2 days later in non-immunized, no adjuvant, and RFA groups. Representative pictures were shown. (**b**) Rectal temperature (Tm) was measured before immunization and 6 and 24 hours post immunization by a Tm probe linked to PhysioSuite (Kent Scientific). (**c**) Serum TNFα and IL-6 levels were measured 24 hours after immunization by commercial ELISA kits (eBiosciences). n=12-14. Representative of two independent experiments. One-way ANOVA with Tukey's multiple comparison test was used to compare differences between groups in **b** and **c**. *, $p<0.05$. NS: Not significant.

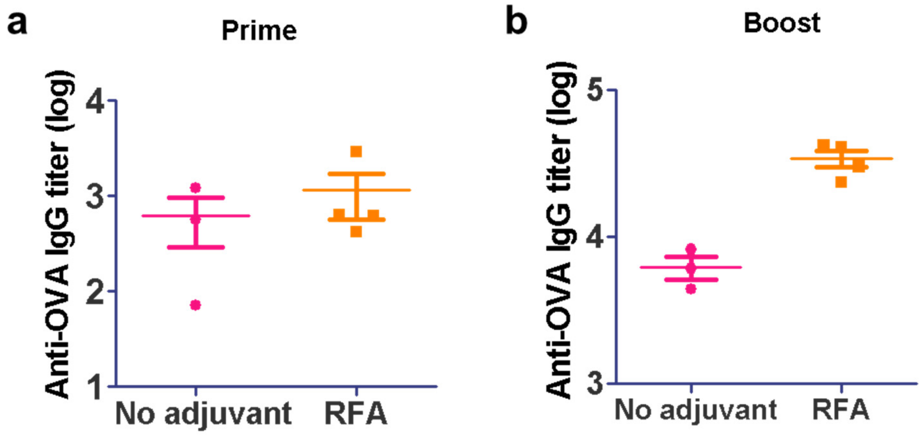

Supplementary Figure 7. Thermal heating is crucial for RFA effects. Lateral back skin of C57BL/6 mice and RF treatment tips were precooled with iced water for 5 minutes. Lateral back skin was then exposed to RF treatment (both precooled) followed by ID injection of 10μg OVA into RF-treated skin. ID injection of the same amount of OVA without precooling was used as control. Immunization was repeated 2 weeks later. Serum anti-OVA antibody titer was measure 2 weeks after prime (**a**) and boost (**b**). n=3-4. Representative of two independent experiments.

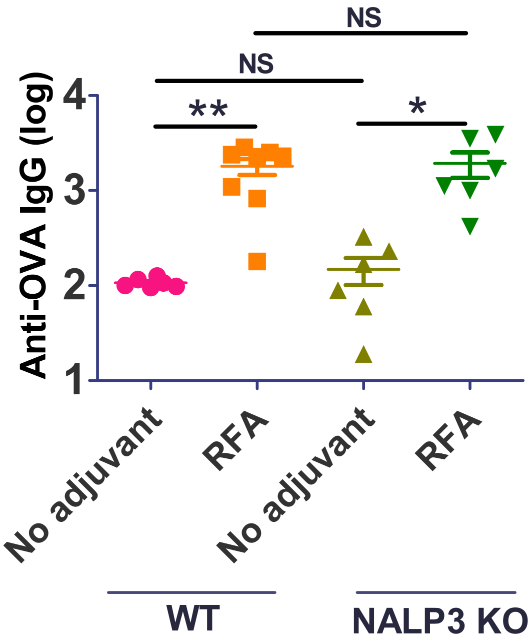

Supplementary Figure 8. NALP3 KO has no significant impact on RFA effects. WT and NALP3 KO mice were exposed to RF or sham treatment followed by ID injection of 10µg OVA into RF or sham-treated skin. Serum anti-OVA antibody titer was measured 2 weeks later. n=6-8. One-way ANOVA with Tukey's multiple comparison test was used to compare differences between groups. *, $p<0.05$; **, $p<0.01$. NS, not significant. Representative of two independent experiments.

Supplementary Table 1. Primer sequences in real-time PCR (5'-3')

Gene	Forward	Reverse
CCL2	TTAAAAACCTGGATCGGAACCAA	GCATTAGCTTCAGATTTACGGGT
CCL7	GATCTCTGCCACGCTTCTGT	ATAGCCTCCTCGACCCACTT
CCL12	GTCCTCAGGTATTGGCTGGA	CACTGGCTGCTTGTGATTCT
CXCL9	GGAGTTCGAGGAACCCTAGTG	GGGATTTGTAGTGGATCGTGC
CXCL12	TGCATCAGTGACGGTAAACCA	TTCTTCAGCCGTGCAACAATC
CHEMERIN	GTGCACAATCAAACCAAACG	GGCAAACTGTCCAGGTAGGA
E-SELECTIN	ATGCCTCGCGCTTTCTCTC	GTAGTCCCGCTGACAGTATGC
IL1β	GCAACTGTTCCTGAACTCAACT	ATCTTTTGGGGTCCGTCAACT
IL6	TAGTCCTTCCTACCCCAATTTCC	TTGGTCCTTAGCCACTCCTTC
IL10	GCTCTTACTGACTGGCATGAG	CGCAGCTCTAGGAGCATGTG
IFNA4 (IFNα)	TGATGAGCTACTACTGGTCAGC	GATCTCTTAGCACAAGGATGGC
IFNβ	CAGCTCCAAGAAAGGACGAAC	GGCAGTGTAACTCTTCTGCAT
IFNγ	ATGAACGCTACACACTGCATC	CCATCCTTTTGCCAGTTCCTC
TNFα	CCCTCACACTCAGATCATCTTCT	GCTACGACGTGGGCTACAG
GAPDH	AGGTCGGTGTGAACGGATTTG	TGTAGACCATGTAGTTGAGGTCA
